# Supplementary material for: Multiple microbial guilds mediate soil methane cycling along a wetland salinity gradient
Source: mSystems. 2024 Jan 3;9(1):e00936-23. doi: 10.1128/msystems.00936-23 (PMC10804969; doi:10.1128/msystems.00936-23)
Supplement: Supplemental Figures — Figures S1 to S13. [file msystems.00936-23-s0001.docx]

**Supplementary Figures for: Multiple microbial guilds mediate soil methane cycling along a wetland salinity gradient**

**Figure S1:** Comparison of methane fluxes obtained in the SF Bay and Delta in our study with those published in a previous meta-analysis (Poffenbarger et al. 2011), shown as smaller gold points plotted at annual scale on the right axis. For this comparison, our hourly flux measurements were also converted to annual estimates based on the formula given by Poffenbarger et al. (2011), and match both left and right scales.

**Figure S2:** Variable importance of soil biogeochemical predictors in LASSO models of soil methane fluxes for (**a**) all soils and (**b**) Delta soils only. Variable importance here is obtained as the model regression coefficients of scaled data, where scaling (by z-scores centered to zero) allows for direct comparison of coefficient weights as “importance.” For all soils (**a**), a training set of 119 soils was used (R^2^ train = 0.628), while a test set of 51 samples produced gave an R^2^ = 0.621. For only Delta soils (**b**), 51 samples were used for the training set (R^2^ = 0.861) and 22 samples were used for the test set (R^2^ = 0.763).

**Figure S3:** Functional genes for element cycling correlated with soil salinity. Genes are color coded by element cycle and pathway as in **Fig. 2**, and selected genes are shown where correlations with salinity had *r* > 0.7. Correlations with salinity are shown in the leftmost heatmap based on all sites, or subsets of sites corresponding to only sites in the Delta, or to only reference wetland sites. The central heatmap shows the relative abundance of these genes across sites, with location indicated by the bar at the top of the heatmap which matches colors in the map of locations in **Fig. 1**.

**Figure S4:** Methane fluxes as a function of selected gene abundances of genes for carbon cycling in Delta soils. Genes are (**a**) xylosidase (*xylB*), (**b**) lignin peroxidase (*ligA*), (**c**) methyl coenzyme M reductase (*mcrA*), (**d**) particulate methane monooxygenase protein C (*pmoC*), (**e**) tungsten-containing formylmethanofuran dehydrogenase subunit F (*fwdF*) and (**f**) phosphotransacetylase (*pta*).

**Figure S5:** Correlations among microbial functions and guilds, salinity, sulfate, and greenhouse gases. Comparisons were made using data from all sites (left column), and only using higher methane, lower salinity sites in the Delta (right column). These comparisons were also made among different annotation approaches, including (**a-b**) gene counts by MG-RAST, (**c-d**) taxonomic assignment of genes annotated by TreeSAPP, and (**e-f**) using 16S rRNA gene taxonomic assignments only. Color bars indicate functions, with colors in a-b corresponding to **Fig. S3**, and c-f corresponding to **Fig. 4**, except red in c-d (HydA - sulfhydrogenase subunit alpha), which was not determined in 16S rRNA gene data. Correlation coefficient values are Pearson’s *r* on log-log data.

**Figure S6:** Principal components analysis of soil microbial community composition (Aitchison’s distance) by 16S rRNA gene, with samples colored based on dominant vegetation type at each site. Vegetation type by itself had a significant effect on microbial assemblages (PERMANOVA R^2^ = 0.38, p = 0.001), as well as after site was taken into account (PERMANOVA R^2^ = 0.06, p = 0.001). Vegetation labels are based on common names. Scientific names are: Cattail -*Typha latifolia*; Tule - *Schoenoplectus acutus;* Three square (ThreeSq) - *Schoenoplectus americanus*; Narrow-leaf cattail (CattailNL) - *Typha angustifolia*; Phragmites (Phrag) - *Phragmites australis*; Pickleweed (PW)- *Salicornia pacifica*; Cordgrass (Cord) - *Spartina foliosa*. Ellipses show 95% confidence intervals around the centroid.

**Figure S7:** Taxonomic composition of sequence reads assigned to cellulose degradation (*cbh-1*, K01225). CPM designates normalized counts per million reads of total sequence belonging to *cbh-1* from each taxonomic group, as determined by taxonomic assignment of these annotations in MG-RAST. The color bar on top corresponds to the wetland sites as in **Fig. 1**.

**Figure S8:** Comparison of sequence abundances of microbial functional guilds determined by 16S rRNA gene taxonomy and shotgun metagenomic sequence annotations filtered by taxonomy using TreeSAPP. Each panel shows log-10 transformed DESeq2 normalized counts per 100,000 reads, with 16S rRNA gene read counts on the x-axis and TreeSAPP read counts on the y-axis. Microbial guilds included (**a**) acetoclastic methanogens here with *Methanosarcinales* included, (**b**) hydrogenotrophic methanogens, (**c**) sulfate reducing bacteria, (**d**) Type I methanotrophs, (**e**) Type IIa methanotrophs, (**f**) Type IIb methanotrophs, (**g**) ammonia oxidizing archaea, (**h**) ammonia oxidizing bacteria, and (**i**) nitrite oxidizing bacteria. Unless otherwise noted, p < 0.001. Derivation of these groups from taxonomic and gene read annotations is detailed in the supplemental text. The dashed line shows a 1:1 line while a solid line is drawn for significant linear regressions.

**Figure S9:** Genera in known microbial guilds strongly correlated (|r| > 0.5) with soil CH_4_ fluxes in the Delta. Color bar at far-left shows guild membership, with guild abbreviations described in **Fig. 4**. Left heatmap shows correlation of each genus with methane fluxes by taxonomic rank. Next, taxonomic membership is shown by major groups (phyla + *Proteobacteria* classes), with colors and groups matching taxa as shown in **Fig. 3**. Right heatmap shows the normalized relative abundance of these genera across soil samples, with wetland location indicated by the color bar above, matching site colors shown in **Fig. 1**.

**Figure S10:** LASSO variable importance in models predicting CH_4_ fluxes in the Delta based off of (**a**) genera (R^2^ = 0.81) associated with known microbial guilds with the greatest absolute variable important scores and (**b**) microbial guild aggregations (R^2^ = 0.65). Guild abbreviations are described in **Fig. 5**. Variable importance here is obtained as the model regression coefficients of scaled data, where scaling (by z-scores centered to zero) allows for direct comparison of coefficient weights as “importance.”

**Figure S11:** Relative abundance in counts per million (CPM) DESeq2 normalized counts of methanotrophic genera across all of the sites, sorted by salinity.

**Figure S12:** Relationships between methanogen: methanotroph ratios and ammonia oxidizers (AO): nitrite oxidizing bacteria (NOB) ratios across all sites (a) and only Delta sites (b). AO is the sum of ammonia oxidizing archaea (AOA) and ammonia oxidizing bacteria (AOB). Lines are linear regression lines with shaded 95% confidence intervals.

**Figure S13**. Correlations between TreeSAPP, *in silico* PCR, and 16S relative abundances for *mcrA* and methanogens (CH4) (a-c) and *pmoA* and methane oxidizing bacteria (MOB) (d-f). TreeSAPP *mcrA* counts in (a) are just for *mcrA* genes assigned taxonomically to methanogens. TreeSAPP *pmoA* counts in (d) are just for *pmoA* genes assigned taxonomically to methane oxidizing bacteria. CPM = counts per million; note that only *in silico* PCR counts are per million unassembled metagenomic reads. Lines are shown for significant linear regression relationships. Note that the R^2^ values are higher for TreeSAPP than for *in silico* PCR.
