# Supplementary material for: Multiple microbial guilds mediate soil methane cycling along a wetland salinity gradient
Source: mSystems. 2024 Jan 3;9(1):e00936-23. doi: 10.1128/msystems.00936-23 (PMC10804969; doi:10.1128/msystems.00936-23)
Supplement: Supplemental Text — More methodological details. [file msystems.00936-23-s0002.docx]

**Supplemental text for: Multiple microbial guilds mediate soil methane cycling along a wetland salinity gradient**

**Contents**

1. Study Sites
2. Soil sampling
3. Greenhouse gas fluxes
4. DNA library construction
5. Sequence classification
6. Guild abundances from 16S rRNA data
7. Guild abundances from shotgun sequence data

**I. Study sites**

Soil samples were obtained from 11 coastal wetland complexes spanning the salinity gradient from the San Francisco Bay to the inland deltaic confluence of the freshwater San Joaquin River and Sacramento River in California (Figure 1). Characteristics of these sites including their geo-location, salinity class, land use, substrate age, and dominant vegetation sampled, along with mean measured salinity, methane and carbon dioxide fluxes are presented in **Table S1**.

Freshwater and oligohaline restored wetland sites (Twitchell Island and Mayberry Farms, respectively) were sampled where greenhouse gas fluxes had previously been intensively characterized (Anderson et al., 2016; Chamberlain et al., 2018, 2019; Hemes et al., 2019; Knox et al., 2015; Miller and Fujii, 2010; Miller et al., 2008; Oikawa et al., 2017; Windham-Myers et al., 2018). Relationships between soil microbes and methane flux had also previously been studied at the Twitchell Island restored wetland (He et al., 2015). Three sampling sites were established at the Mayberry Farms wetland to capture variability in salinity across the site. These sites were paired with freshwater and brackish reference wetlands (Sand Mound Slough and Brown’s Island, respectively), used as baseline references given the lack of historic agricultural activity on these isolated islands (Vasey et al., 2012; Watson and Byrne, 2009) with undisturbed ca. 6000-year-old peat soils (Drexler et al., 2009). The undisturbed soils of the Joice Island mesohaline reference wetland (Byrne et al., 2001; Ferner, 2011; Smith and Kelt, 2019) were compared to nearby mesohaline wetlands with mixed land use history at the Rush Ranch National Estuarine Research Reserve (Callaway et al., 2011; Ferner, 2011; Vasey et al., 2012; Whitcraft et al., 2011), including “historic sites” fringing a large tidal creek (Rush Ranch SE), and high marsh plain (Rush Ranch oxbow), along with a muted tidal marsh impoundment classified with restored wetlands (Rush Ranch managed). No appropriate reference wetland sites were accessible to represent the meso – polyhaline wetland transition (ca. 10-25 PSU salinity), although three wetland sites which were either tidally breached or restored were sampled in this range, including the Goodyear Slough (Smith and Kelt, 2019; Smith et al., 2018), White Slough (Williams and Orr, 2002), and Tolay Creek Wetlands (Williams and Faber, 2001; Williams and Orr, 2002). Full seawater salinity (polyhaline) sites included the Muzzi Marsh restored wetland (Callaway et al., 2011; Williams and Faber, 2001; Williams and Orr, 2002), and two locations in the undisturbed reference wetlands at the China Camp National Estuarine Research Reserve (Baye, 2012; Callaway et al., 2011; Ferner, 2011; Malamud-Roam and Ingram, 2001; Vasey et al., 2012), where soils have been dated to ca. 5000 years before present (Goman et al., 2008).

**II. Soil sampling**

At each sampling location, dominant plant species were identified by percentage land cover and between one and three plant species were selected to target for nearby soil cores. Each plant type was sampled at three coring locations (A, B, C) for replicate analyses. Sampling consisted of retrieving intact soil cores with a Split Core Sampler fitted with an auger tip (AMS Inc., American Falls, ID). Soil cores were split into a 0-5 cm (D1) and a 5-15 cm (D2) section (0 cm corresponding to the core top), and each section was individually homogenized in a clean tray and large roots were removed. Approximately 50g of homogenized soil material was collected in a 50ml Falcon tube for DNA analyses and immediately frozen on dry ice before being transferred to a -80°C freezer. An additional intact soil core was retrieved adjacent to the DNA soil core and transported to the lab at ambient temperature for greenhouse gas analyses and soil geochemistry. Porewater was collected from PVC sampling pipes slotted at 5-10 cm beneath the soil surface using a hand pump to extract 40 ml water samples, which were filtered (0.45 um syringe filter) and frozen for subsequent analyses. *In situ* measurements of water pH, temperature, conductivity, dissolved oxygen, and reduction-oxidation (redox) potential were collected using a YSI Multi-Parameter Water Quality Sonde (Model 6920-v2; YSI Inc., Yellow Springs, OH, USA).

**III. Greenhouse gas fluxes**

Intact soil cores were analyzed for greenhouse gas production (CH_4_, CO_2_, and H_2_O) using a Los Gatos Research Greenhouse Gas Analyzer (GGA, Los Gatos Research, Mountain View, CA). The GGA measures CO_2_ and CH_4_ concentrations at 1 Hz (every second) using tunable laser cavity ringdown spectroscopy, with a stated precision of < 2 ppb (1σ @ 1 Hz) over an operating range of 0.1 to 100 ppm. Cores were closed on the bottom with airtight caps and loaded into a 2 L glass Mason jar fitted with airtight Bev-A-Line IV connective tubing (US Plastic Corp, Lima OH) that allowed continuous gas exchange with the GGA’s pumped internal chamber. Soil core fluxes were measured over two consecutive 500 second intervals and ventilated between these cycles for 100 s by gently agitating air above the opened jar while the GGA’s pump purged the gas lines and chamber with ambient air. Time series data on CO_2_ and CH_4_ from the (linear slope) second measurement interval were used to calculate rates of concentration increases within the experimental chamber. Rates of GHG fluxes (mmol/m^2^/s and mmol/m^2^/h equivalents) were calculated by accounting for the empty volume of the experimental chamber and tubing, and surface area of the soil core through which gases passed.

**IV. DNA library construction**

Frozen soil samples were thawed at 4°C, homogenized, and approximately 0.5 g of soil sample (by wet weight) was removed for DNA extraction. Both the 0-5 cm (D1) and 5-15 cm (D2) soil core fractions were extracted. DNA extractions were performed with the PowerLyzer PowerSoil DNA isolation kit (Mo Bio Laboratories, Inc., Carlsbad, CA) according to the manufacturer’s instructions. DNA yield was assessed with the Qubit 2.0 Fluorometer (Invitrogen, Carlsbad, CA, USA).

To determine microbial community composition, we amplified the V4 region of the 16S rRNA gene using barcoded primers 515 F (5′-GTGCCAGCMGCCGCGGTAA-3′) and 806 R (5′-GGACTACHVGGGTTCTAAT-3′) established by Caporaso et al. (2012). DNA amplicon generation of the V4 region was performed using Quantabio 5PRIME HotMasterMix (Quantabio, Beverly, MA). The forward primer consisted of the Illumina adapter sequence (5’-AATGATACGGCGACCACCGAGATCTACAC-3’) attached to the Read 1 sequencing primer binding site (5’-TCTTTCCCTACA-3’) followed by 0-5 random bases then established primer 515F. Additional details describing staggered barcodes are in Tremblay et al. (2015), and PCR amplification conditions described in Hartman et al. (2017). Pooled amplicons were purified with the Agencourt AMPure XP purification system (Beckman Coulter, Brea, CA, USA) and analyzed with an Agilent Bioanalyzer 2100 (Agilent Technologies, Palo Alto, CA, USA) to confirm appropriate amplicon size. Before sequencing, a PhiX spike-in shotgun library was added to the amplicon pool for a final concentration of about 2-25%. Amplicon sequencing was performed according to the JGI’s standard protocols: amplicons were diluted to 10 nM, quantified by quantitative PCR, and sequenced on the Illumina MiSeq platform (2x300bp, reagent kit v.3; Illumina Inc., Carlsbad, CA, USA).

Soil metagenomic shotgun sequence data were obtained using a 96 well plate-based DNA library preparation for Illumina sequencing, performed on the PerkinElmer Sciclone NGS liquid handling system with a Kapa Biosystems library preparation kit. 200 ng of sample DNA was sheared to 300 bp using a Covaris LE220 focused-ultrasonicator. Sheared DNA fragments were subjected to two rounds of size selection with Solid Phase Reversible Immobilization (SPRI) beads (Beckman Coulter) and selected fragments were end-repaired, A-tailed, and ligated with Illumina compatible sequencing adaptors from IDT containing a unique index barcode for each sample library. These libraries were then quantified using KAPA Biosystems’ next-generation sequencing library qPCR kit and run on a Roche LightCycler 480 real-time PCR instrument. Quantified libraries were multiplexed, and the pooled libraries were prepared for sequencing utilizing a TruSeq paired-end cluster kit, v4, and Illumina’s cBot instrument to generate a clustered Illumina HiSeq sequencing flow cell.

Sequencing of the flow cell was performed on the Illumina HiSeq2500 sequencer at the JGI using HiSeq TruSeq SBS v.4 sequencing kits using a 2 x150 or 2 x 250 run mode. Overall, sequencing libraries yielded ~5.3 Gbp per sample after contaminant and quality filtering.

**V. DNA sequence classification**

16S rRNA gene amplicon sequences were analyzed using the iTagger v.1.1 pipeline developed at the Joint Genome Institute (JGI) and described by Tremblay et al., (2015). The pipeline removes Illumina adapters and PhiX sequences, and performs paired-end read assembly, read quality filtering, chimera-checking, and read clustering into operational taxonomic units (OTUs) at 97% similarity. Taxonomic classification of OTUs was achieved using the ‘assignTaxonomy’ function in the *dada2* R package (Callahan et al. 2016), with the SILVA version 138.1 reference database (Quast et al. 2013).

Shotgun sequence annotations were generated from FASTQ-formatted sequencing read data from each sample with the MG-RAST metagenome annotation server (Meyer et al., 2008; Wilke et al., 2016). Briefly, the MG-RAST pipeline annotates shotgun metagenome sequence data by clustering translated protein sequence data using CD-HIT (Fu et al., 2012), then matching protein sequence clusters to the M5nr database of non-redundant genomic protein sequences (Wilke et al., 2012) using the BLAT algorithm (Kent, 2002). Counts of functional annotations organized by the KEGG Orthology (Kanehisa et al., 2016) were downloaded for each sample from the MG-RAST API (Wilke et al., 2016) using a custom Python script which merged annotations into a single table of counts for each KO for each sample.

**VI. Functional guild assignment from 16S rRNA gene taxonomic group associations**

Microbial functional guilds were assigned based on 16S rRNA gene taxonomic identities where biogeochemical functions could be ascertained from taxa names, including guilds for methanogenesis, oxidation of methane, ammonia, and nitrite, reduction of sulfate, and reduction or oxidation of iron. While we recognize the inherent limitations of this approach, these functions were assigned where literature reviews indicated functions are relatively monophyletic, or where taxonomic naming conventions are applied consistently. Guild assignments were applied based on taxonomic strings in OTU abundance tables, filtering only organisms matching search terms using grep searches for particular taxa names. Search terms and supporting literature for each microbial guild are described in detail below, and the code to derive these guild abundances from OTU tables is available on [GitHub](https://github.com/cliffbueno/SF_microbe_methane/blob/main/modules/3_OTU_subsetting_modules_v.0.4_strip.r) (‘Get_16S_guilds_alt’ function in section 3 of this code).

*Methane production and oxidation*

Anaerobic methanogenesis is a monophyletic function carried out exclusively by archaeal taxonomic groups. Abundances of methanogens were obtained by filtering OTU tables according to assigned taxonomy, using the search term “Methano” across taxonomic ranks. This naming prefix appears present in dominant methanogen classes (*Methanobacteria*, *Methanococci*, *Methanopyri*), orders, families, and genera (Hedderich and Whitman 2013, Nazaries et al. 2013). This search was further updated to remove Methanoperedenaceae, Syntrophoarchaeaceae, and ANME, which are methane oxidizers containing “Methano” in some of their taxonomic levels, and adding “Methermicoccaceae” which are methanogens without “Methano” in the name. Within results for methanogenic taxa, a further search was conducted to split taxa into acetoclasts (“Methanosaetaceae” and “Methanotrichaceae”), mixotrophs (“Methanosarcinaceae” and “Methanobacteriaceae”), methyl-reducers (“Methanomasiliicoccales” and “Methanonatronarchaeales”); and all other groups were labeled as “hydrogenotrophic.” We recognize this classification scheme is an approximation based on current knowledge, and that lack of classification to family or order level will result in some misclassifications.

Methanotrophic microbial groups were identified from published reviews and divided into functional guilds based on monophyletic taxonomic groupings (Hedderich and Whitman 2013, Nazaries et al. 2013). Nearly all genera of methanotrophs have names beginning with the string “Methylo” (Knief 2015, Khadka et al. 2018), which was used to filter (grep) taxonomic abundance tables for methanotrophs. A notable exception to this naming convention is *Verrucomicrobia* organisms, named with the prefix “Methyla,” which would not be captured by the initial search term. Guilds of methanotrophic taxa were delineated by further taxonomic subdivision, with search filtering for class-level taxa “Gammaproteobacteria” used to identify Type I methanotrophs, and “Alphaproteobacteria” used to filter Type II methanotrophs, which were further subdivided to separate Type IIa methanotrophs using the search

term “Beijerinckiaceae” on family-level taxonomic assignments (Knief 2015, Khadka et al. 2018). A further class level filter for “Betaproteobacteria” was used to remove methylotrophs from the original “Methylo” search, as this class contains true methylotrophs (e.g., utilizing methanol) like those in the family *Methylophilaceae* (Chistoserdova 2015, Chistoserdova et al. 2009, Smith and Wrighton 2019). Finally, methane consumption may also be carried out by anaerobic methane-oxidizing archaea, which follow a taxonomic naming convention of “ANME” (Wang et al. 2014) which was used as an additional search filter.

*Nitrifiers and anammox*

Some microbial nitrogen cycling functions like nitrification and anamox (anaerobic ammonia oxidation) appear to be phylogenetically constrained. Nitrifying bacteria can be classified into functional guilds including ammonia oxidizers (including bacteria and archaea, or AOB and AOA), nitrite oxidizers (NOB), and anammox bacteria (in the *Planctomycetes*) (Bouskill et al., 2012). Significantly, nitrifying bacteria follow a taxonomic convention where nitrite oxidizers are named with the prefix “Nitro” and ammonia oxidizers with the prefix “Nitroso”, except for anammox bacteria. This naming convention is broadly applicable to AOA (Alves et al., 2018), AOB, and NOB (Bouskill et al., 2012), although it may not capture certain Actinobacterial AOB (Khadka et al. 2018). The search term “Nitros” was first used to obtain nitrifying bacteria and archaea, which were then split using an additional search for “Nitroso” to separate ammonia oxidizers. An additional “Archaea” search term at domain level taxonomy was applied to separate AOB from AOA. Anammox bacteria were obtained by searching for genus names of the few confirmed genera, including “Kuenenia”, “Anammoxoglobus”, “Scalindua”, “Brocadia”, and “Jettenia” (Cai et al. 2020).

*Sulfate reduction, oxidation, and syntrophs*

Sulfate reducing organisms were identified following published comparative analyses and reviews (Plugge et al. 2011, Müller et al. 2015), and enumerated using grep-based filtering of taxonomic abundance (OTU) tables. Many sulfate reducers have taxa names beginning with “Desulf,” which was used as the primary search (grep) term. However, not all organisms with these names in taxonomic strings are sulfate reducers, and organisms containing the strings “Nitros” (*Nitrospinaceae*), “Syntroph” and “Geobacter” (both treated as separate guilds) were removed from the initial search results. On the other hand, several other taxa known to reduce sulfate do not indicate this term in their names, including bacteria and archaea. Additional search terms (Müller et al. 2015) included "Caldiserica”, “Thermanaeromonas”, “Sporomusaceae”, “Carboxydothermus”, “Pelotomaculum”, “Moorella”, “Ammonifex”, “Acetonema”, “Thermosinus”, “Thermanaeromonas”, “Carboxydothermus”, “Caldiserica”, “Gordonibacter”, “Thermodesulfobium”, “Thermodesulfovibrio”, and “Magnetobacterium”, along with the sulfate reducing archaea “Archaeoglobus”, “Pyrobaculum”, “Vulcanisaeta”, and “Caldirvirga”.

Sulfur oxidizing organisms were identified following (Müller et al. 2015), with many organisms containing a taxonomic string “Thio.” Other organisms not containing this name were obtained using the search (grep) terms “Allochromatium”, “Marichromatium”, “Halochromatium”, “Alkalilimnicola”, “Halorhodospira”, “Ruthia”, “Vesicomyosocius”, “Sedimenticola”, “Sideroxydans”, “Sulfuricella”, “Riegeria”, “Azospirillum”, “Rhodomicrobium”, “Magnetospirillum”, “Magnetococcus”, “Chlorobium”, “Chlorobaculum”, and “Prosthecochloris".

Syntrophic bacteria are closely related to sulfate reducers, though some may have lost the capability for sulfate reduction (Plugge et al. 2011). The search term “Syntroph” was used to identify these organisms, given many are found in the order *Syntrophobacterales*, with three families (Plugge et al. 2011). However, other groups of syntrophic bacteria may exist which would not be obtained by this search (Sieber et al. 2012, Worm et al. 2014). Furthermore, this does not take into account taxa that may have alternative lifestyles without syntrophy.

*Iron reduction and oxidation*

Iron reducing bacteria were enumerated by a search filter for common environmental iron reducers, using the terms “Geobacter”, “Shewanella”, “Thermoanaerobacter”, “Deferribacter”, “Geothrix”, “Albidiferax”, and “Deferrisomatota”. Notably, this is not a comprehensive list of taxa capable of iron reduction (Schröder et al. 2003, Hori et al. 2015). Iron oxidizing bacteria were identified using search terms obtained from review papers (Hedrich et al. 2011, Ilbert and Bonnefoy 2013, Kato et al. 2015), including the common name fragments “Ferro”, “Lepto”, and “Metallo”, along with additional taxa including “Mariprofundus”, “Gallionella”, “Sideroxydans”, “Acidithiobacillus”, “Rhodopseudomonas”, and “Sulfolobus."

**VII. Functional guild abundances from shotgun sequence data**

The abundances of microbial functional guilds for methanogens, methanotrophs, sulfate reducers, ammonia oxidizers, nitrite oxidizing bacteria, and microbial single copy marker genes were determined from shotgun sequencing data using TreeSAPP (Morgan-Lang et al., 2020), then compared to the abundances of similar groups derived from 16S rRNA gene taxonomy (described below). TreeSAPP enables taxonomic classification of functional gene reads needed to obtain microbial guilds where gene function may be altered by taxonomic position. At a high level, the TreeSAPP pipeline compares query reads to pre-compiled models for each gene, places reads into pre-calculated phylogenetic trees, and determines taxonomy or functional clade placement by least common ancestor (LCA) (Hanson et al., 2016). Reference sequences are obtained from existing public data sources and aligned using MAFFT (Katoh and Standley, 2013), with alignments used to build both phylogenetic trees using RAxML (Berger and Stamatakis, 2011) or FastTree (Price et al., 2010), and hidden Markov models (HMMs) using HMMer v3.1 (Eddy, 1998). Query sequences were assigned taxonomy and functional phylogeny by the TreeSAPP pipeline by first identifying ORFs using Prodigal (Hyatt et al., 2010), then identifying homology to target profiles to which sequences were aligned (using hmmsearch and hmmalign in HMMer). Aligned sequences were next assigned phylogenetic placement in reference trees using the RAxML evolutionary placement algorithm (Berger and Stamatakis, 2011), and assigned to taxonomic and functional groups using LCA.

This method was applied to several genes relevant to derivation of functional guilds in soils, including *mcrABG* (methanogen guilds), *dsrAB* (sulfate reducers and oxidizers), *amo/pmoABC* (ammonia oxidizers and methanotrophs), and *nxrAB* (nitrite oxidizers), as well as several single copy phylogenetic markers for DNA replication (*recA*, *rpoB*, *RPS3A)*. Reference gene reads and functional group assignments were obtained from multiple sources as described in the original TreeSAPP publication (Morgan-Lang et al., 2020). An initial goal was to normalize functional guild counts from TreeSAPP using single copy marker DNA replication genes. However, counts of these marker genes and guild-related genes were quite low in shotgun sequence data, prompting a decision to instead use DESeq2 normalization (Love et al., 2014) of guild counts in the same manner as applied to 16S rRNA gene-based guilds counts.

The TreeSAPP commands used to create reference packages (with `treesapp create`) included the following arguments: *--trim_align* to trim the multiple sequence alignment with BMGE prior to inferring a reference phylogeny; *--fast* to use FastTree for inferring the phylogeny; *--cluster* and *-p* *<sequence identity>* to dereplicate the input sequences, reducing the size of the reference phylogeny and thereby increase phylogenetic placement speed while maintaining diversity; *--screen 'Bacteria,Archaea'* to remove sequences derived from Eurkaryotes; *--min_taxonomic_rank c* to ensure all reference sequences have a taxonomic lineage resolved to at least the class rank; *--profile <hmm file>* to use homologous sequences identified by hidden Markov model (HMM) alignment in cases where curation was deemed insufficient or protein families contained accessory domains that interfered with alignment and phylogenetic inference. This was the case for NxrA (COG5013), RecA/RadA (COG0468), RpoB (COG0085), and DsrAB (COG2221), for which HMMs were downloaded from EggNOG v4.5. An HMM profile wasn't necessary to help curate McrA and XmoA sequences as these were manually reviewed. The `treesapp assign` commands for identifying sequencing reads homologous to each reference package used the default parameters. The parameter defaults related to sequence processing and filtering required HMM sequence alignments to have a maximum E-value of 1E-5 and a minimum bit score of 20, and placement likelihoods (computed by RAxML) needed to exceed 0.1. No multiple sequence alignment trimming was done with BMGE prior to sequence placement.

**References**

Alves, R.J.E., Minh, B.Q., Urich, T., von Haeseler, A., and Schleper, C. (2018). Unifying the global phylogeny and environmental distribution of ammonia-oxidising archaea based on amoA genes. Nat Commun *9*, 1517.

Anderson, F.E., Bergamaschi, B., Sturtevant, C., Knox, S., Hastings, L., Windham-Myers, L., Detto, M., Hestir, E.L., Drexler, J., Miller, R.L., et al. (2016). Variation of energy and carbon fluxes from a restored temperate freshwater wetland and implications for carbon market verification protocols. Journal of Geophysical Research: Biogeosciences *121*, 777–795.

Baye, P.R. (2012). Tidal Marsh Vegetation of China Camp, San Pablo Bay, California. San Francisco Estuary and Watershed Science *10*.

Berger, S.A., and Stamatakis, A. (2011). Aligning short reads to reference alignments and trees. Bioinformatics *27*, 2068–2075.

Bouskill, N.J., Tang, J., Riley, W.J., and Brodie, E.L. (2012). Trait-based representation of biological nitrification: model development, testing, and predicted community composition. Front. Microbio. *3*, 364.

Brandmaier, A.M., Prindle, J.J., Mcardle, J.J., and Lindenberger, U. (2016). Theory-guided exploration with structural equation model forests. Psychological Methods *21*, 566–582.

Bräuer, S.L., Basiliko, N., M. P. Siljanen, H., and H. Zinder, S. (2020). Methanogenic archaea in peatlands. FEMS Microbiology Letters *367*, fnaa172.

Byrne, R., Ingram, B.L., Starratt, S., Malamud-roam, F., Collins, J.N., and Conrad, M.E. (2001). Carbon-isotope, diatom, and pollen evidence for Late Holocene salinity change in a brackish marsh in the San Francisco Estuary. Quaternary Research *55*, 66–76.

Cai, M., Ye, F., Wu, J., Wu, Q., Wang, Y., and Hong, Y. (2020). Bias of marker genes in PCR of anammox bacteria in natural habitats. PLoS ONE *15*, e0239736.

Callahan BJ, McMurdie PJ, Rosen MJ, Han AW, Johnson AJA, Holmes SP. DADA2: High-resolution sample inference from Illumina amplicon data. *Nat Methods* 2016; **13**: 581–583.

Callaway, J.C., Parker, T.V., and Vasey, M.C. (2011). Tidal wetland restoration in San Francisco Bay: history and current issues. San Francisco Estuary and Watershed Science *9*.

Chamberlain, S.D., Anthony, T.L., Silver, W.L., Eichelmann, E., Hemes, K.S., Oikawa, P.Y., Sturtevant, C., Szutu, D.J., Verfaillie, J.G., and Baldocchi, D.D. (2018). Soil properties and sediment accretion modulate methane fluxes from restored wetlands. Global Change Biology *24*, 4107–4121.

Chamberlain, S.D., Hemes, K.S., Eichelmann, E., Szutu, D.J., Verfaillie, J.G., and Baldocchi, D.D. (2019). Effect of drought-induced salinization on wetland methane emissions, gross ecosystem productivity, and their interactions. Ecosystems 1–14.

Chistoserdova, L. (2015). Methylotrophs in natural habitats: current insights through metagenomics. Applied Microbiology and Biotechnology *99*, 5763–5779.

Chistoserdova, L., Kalyuzhnaya, M.G., and Lidstrom, M.E. (2009). The expanding world of methylotrophic metabolism. Annu. Rev. Microbiol. *63*, 477–499.

DeSantis, T.Z., Hugenholtz, P., Larsen, N., Rojas, M., Brodie, E.L., Keller, K., Huber, T., Dalevi, D., Hu, P., and Andersen, G.L. (2006). Greengenes, a Chimera-Checked 16S rRNA Gene Database and Workbench Compatible with ARB. Applied and Environmental Microbiology *72*, 5069–5072.

Drexler, J.Z., de Fontaine, C.S., and Brown, T.A. (2009). Peat accretion histories during the past 6,000 years in marshes of the Sacramento – San Joaquin Delta, CA, USA. Estuaries and Coasts *32*, 871–892.

Eddy, S.R. (1998). Profile hidden Markov models. Bioinformatics *14*, 755–763.

Fan, Y., Chen, J., Shirkey, G., John, R., Wu, S.R., Park, H., and Shao, C. (2016). Applications of structural equation modeling (SEM) in ecological studies: an updated review. Ecological Processes *5*, 19.

Ferner, M.C. (2011). A Profile of the San Francisco Bay National Estuarine Research Reserve (San Francisco, CA).

Fu, L., Niu, B., Zhu, Z., Wu, S., and Li, W. (2012). CD-HIT: accelerated for clustering the next-generation sequencing data. Bioinformatics *28*, 3150–3152.

Goman, M., Malamud-Roam, F., and Ingram, B.L. (2008). Holocene environmental history and evolution of a tidal salt marsh in San Francisco Bay, California. Journal of Coastal Research *24*, 1126–1137.

Grace, J.B., Anderson, T.M., Olaf, H., and Scheiner, S. (2010). On the specification of structural equation models for ecological systems. Ecological Monographs *80*, 67–87.

Hanson, N.W., Konwar, K.M., and Hallam, S.J. (2016). LCA*: an entropy-based measure for taxonomic assignment within assembled metagenomes. Bioinformatics *32*, 3535–3542.

He, S., Malfatti, S.A., McFarland, J.W., Anderson, F.E., Pati, A., Huntemann, M., Tremblay, J., Glavina, T., Waldrop, M.P., Windham-Myers, L., et al. (2015). Patterns in wetland microbial community composition and functional gene repertoire associated with methane emissions. MBio *6*, e00066-15.

Hedderich, R., and Whitman, W.B. (2013). Physiology and biochemistry of the methane-producing archaea. In The Prokaryotes, E. Rosenberg, E.F. DeLong, S. Lory, E. Stackebrandt, and F. Thompson, eds. (Berlin, Heidelberg: Springer Berlin Heidelberg), pp. 635–662.

Hedrich, S., Schlömann, M., and Johnson, D.B. (2011). The iron-oxidizing proteobacteria. Microbiology *157*, 1551–1564.

Hemes, K.S., Chamberlain, S.D., Eichelmann, E., Anthony, T., Valach, A., Kasak, K., Szutu, D., Verfaillie, J., Silver, W.L., and Baldocchi, D.D. (2019). Assessing the carbon and climate benefit of restoring degraded agricultural peat soils to managed wetlands. Agricultural and Forest Meteorology *268*, 202–214.

Hori, T., Aoyagi, T., Itoh, H., Narihiro, T., Oikawa, A., Suzuki, K., Ogata, A., Friedrich, M.W., Conrad, R., and Kamagata, Y. (2015). Isolation of microorganisms involved in reduction of crystalline iron(III) oxides in natural environments. Front. Microbiol. *6*.

Hyatt, D., Chen, G.-L., LoCascio, P.F., Land, M.L., Larimer, F.W., and Hauser, L.J. (2010). Prodigal: prokaryotic gene recognition and translation initiation site identification. Bioinformatics *11*, 119.

Ilbert, M., and Bonnefoy, V. (2013). Insight into the evolution of the iron oxidation pathways. Biochimica et Biophysica Acta (BBA) - Bioenergetics *1827*, 161–175.

Jacobucci, R. (2017). regsem: regularized structural equation modeling. ArXiv 1703.08489.

Jacobucci, R., Grimm, K.J., and McArdle, J.J. (2016). Regularized structural equation modeling. Structural Equation Modeling *23*, 555–566.

Kanehisa, M., Sato, Y., Kawashima, M., Furumichi, M., and Tanabe, M. (2016). KEGG as a reference resource for gene and protein annotation. Nucleic Acids Research *44*, D457–D462.

Kato, S., Ohkuma, M., Powell, D.H., Krepski, S.T., Oshima, K., Hattori, M., Shapiro, N., Woyke, T., and Chan, C.S. (2015). Comparative genomic insights into ecophysiology of neutrophilic, microaerophilic iron oxidizing bacteria. Front. Microbiol. *6*, 1265.

Katoh, K., and Standley, D.M. (2013). MAFFT multiple sequence alignment software version 7: improvements in performance and usability. Molecular Biology and Evolution *30*, 772–780.

Kent, W.J. (2002). BLAT — The BLAST-Like Alignment Tool. Genome Research *12*, 656–664.

Khadka, R., Clothier, L., Wang, L., Lim, C.K., Klotz, M.G., and Dunfield, P.F. (2018). Evolutionary history of copper membrane monooxygenases. Frontiers in Microbiology 2493.

Knief, C. (2015). Diversity and habitat preferences of cultivated and uncultivated aerobic methanotrophic bacteria evaluated based on pmoA as molecular marker. Frontiers in Microbiology *6*, 1346.

Knox, S.H., Sturtevant, C., Matthes, J.H., Koteen, L., Verfaille, J., and Baldocchi, D. (2015). Agricultural peatland restoration: effects of land-use change on greenhouse gas (CO2 and CH4) fluxes in the Sacramento-San Joaquin Delta. Global Change Biology *21*, 750–765.

Love, M.I., Huber, W., and Anders, S. (2014). Moderated estimation of fold change and dispersion for RNA-seq data with DESeq2. Genome Biology *15*, 550.

Malamud-Roam, A.F., and Ingram, B.L. (2001). Carbon isotopic compositions of plants and sediments of tide marshes in the San Francisco Estuary. Journal of Coastal Research *17*, 17–29.

Meyer, F., Paarmann, D., D’Souza, M., Olson, R., Glass, E.M., Kubal, M., Paczian, T., Rodriguez, A., Stevens, R., Wilke, A., et al. (2008). The metagenomics RAST server – a public resource for the automatic phylogenetic and functional analysis of metagenomes. BMC Bioinformatics *9*, 386.

Miller, R.L., and Fujii, R. (2010). Plant community, primary productivity, and environmental conditions following wetland re-establishment in the Sacramento-San Joaquin Delta, California. Wetland Ecology and Management *18*, 1–16.

Miller, R.L., Fram, M., Fujii, R., and Wheeler, G. (2008). Subsidence reversal in a re-established wetland in the Sacramento-San Joaquin Delta, California, USA. San Francisco Estuary and Watershed Science *6*.

Morgan-Lang, C., McLaughlin, R., Armstrong, Z., Zhang, G., Chan, K., and Hallam, S.J. (2020). TreeSAPP: the Tree-based Sensitive and Accurate Phylogenetic Profiler. Bioinformatics *36*, 4706–4713.

Müller, A.L., Kjeldsen, K.U., Rattei, T., Pester, M., and Loy, A. (2015). Phylogenetic and environmental diversity of DsrAB-type dissimilatory (bi)sulfite reductases. ISME J *9*, 1152–1165.

Nazaries, L., Murrell, J.C., Millard, P., Baggs, L., and Singh, B.K. (2013). Methane, microbes and models: fundamental understanding of the soil methane cycle for future predictions: Methane, microbes and models. Environ Microbiol *15*, 2395–2417.

Oikawa, P.Y., Jenerette, G.D., Knox, S.H., Sturtevant, C., Verfaillie, J.G., Dronova, I., Poindexter, C.M., Eichelmann, E., and Baldocchi, D.D. (2017). Evaluation of a hierarchy of models reveals importance of substrate limitation for predicting carbon dioxide and methane exchange in restored wetlands. Journal of Geophysical Research: Biogeosciences *122*, 145–167.

Plugge, C.M., Zhang, W., Scholten, J.C.M., and Stams, A.J.M. (2011). Metabolic flexibility of sulfate-reducing bacteria. Frontiers in Microbiology *2*, 81.

Price, M.N., Dehal, P.S., and Arkin, A.P. (2010). FastTree 2 – approximately maximum-likelihood trees for large alignments. PLOS ONE *5*, e9490.

Quast C, Pruesse E, Yilmaz P, Gerken J, Schweer T, Yarza P, et al. The SILVA ribosomal RNA gene database project: improved data processing and web-based tools. *Nucleic Acids Res* 2013; **41**: D590–D596.Rosseel, Y. (2012). lavaan: an R package for structural equation modeling. Journal of Statistical Software *48*, 1–36.

Schröder, I., Johnson, E., and de Vries, S. (2003). Microbial ferric iron reductases. FEMS Microbiol Rev *27*, 427–447.

Sieber, J.R., McInerney, M.J., and Gunsalus, R.P. (2012). Genomic insights into ayntrophy: the paradigm for anaerobic metabolic cooperation. Annual Review of Microbiology 429–452.

Smith, G.J., and Wrighton, K.C. (2019). Metagenomic approaches unearth methanotroph phylogenetic and metabolic diversity. In Methylotrophs and Methylotroph Communities, (Caister Academic Press), pp. 57–84.

Smith, K.R., and Kelt, D.A. (2019). Waterfowl management and diet of the salt marsh harvest mouse. The Journal of Wildlife Management *83*, 1687–1699.

Smith, K.R., Riley, M.K., Thompson, L., Woo, I., Statham, M.J., Estrella, S., and Kelt, D.A. (2018). Toward salt marsh harvest mouse recovery: a review. San Francisco Estuary and Watershed Science *16*.

Tremblay, J., Singh, K., Fern, A., Kirton, E.S., He, S., Woyke, T., Lee, J., Chen, F., Dangl, J.L., and Tringe, S.G. (2015). Primer and platform effects on 16S rRNA tag sequencing. Frontiers in Microbiology *6*, 771.

Vasey, M.C., Parker, V.T., and Callaway, J.C. (2012). Tidal wetland vegetation in the San Francisco Bay-Delta Estuary. San Francisco Estuary and Watershed Science *10*.

Wang, F.-P., Zhang, Y., Chen, Y., He, Y., Qi, J., Hinrichs, K.-U., Zhang, X.-X., Xiao, X., and Boon, N. (2014). Methanotrophic archaea possessing diverging methane-oxidizing and electron-transporting pathways. ISME J *8*, 1069–1078.

Wang, Q., Garrity, G.M., Tiedje, J.M., and Cole, J.R. (2007). Naive Bayesian classifier for rapid assignment of rRNA sequences into the new bacterial taxonomy. Applied and Environmental Microbiology *73*, 5261–5267.

Watson, E.B., and Byrne, R. (2009). Abundance and diversity of tidal marsh plants along the salinity gradient of the San Francisco Estuary: implications for global change ecology. Plant Ecology *205*, 113–128.

Whitcraft, C.R., Grewell, B.J., and Baye, P.R. (2011). Estuarine vegetation at Rush Ranch Open Space Preserve, San Francisco Bay National Estuarine Research Reserve, California. San Francisco Estuary and Watershed Science *9*.

Wilke, A., Harrison, T., Wilkening, J., Field, D., Glass, E.M., Kyrpides, N., Mavrommatis, K., and Meyer, F. (2012). The M5nr: a novel non-redundant database containing protein sequences and annotations from multiple sources and associated tools. BMC Bioinformatics *13*, 141.

Wilke, A., Bischof, J., Gerlach, W., Glass, E., Harrison, T., Keegan, K.P., Paczian, T., Trimble, W.L., Bagchi, S., Grama, A., et al. (2016). The MG-RAST metagenomics database and portal in 2015. Nucleic Acids Research *44*, D590–D594.

Williams, P., and Faber, P. (2001). Salt marsh restoration experience in San Francisco Bay. Journal of Coastal Research *27*, 203–211.

Williams, P.B., and Orr, M.K. (2002). Physical evolution of restored breached levee salt marshes in the San Francisco Bay Estuary. Restoration Ecology *10*, 527–542.

Windham-Myers, L., Bergamaschi, B., Anderson, F., Knox, S., Miller, R., and Fujii, R. (2018). Potential for negative emissions of greenhouse gases (CO2, CH4 and N2O) through coastal peatland re-establishment: novel insights from high frequency flux data at meter and kilometer scales. Environmental Research Letters *13*, 045005.

Worm, P., Koehorst, J.J., Visser, M., Sedano-Nunez, V.T., Shaap, P.J., Plugge, C.M., Sousa, D.Z., and Stams, A.J.M. (2014). A genomic view on syntrophic versus non-syntrophic lifestyle in anaerobic fatty acid degrading communities. Biochimica et Biophysica Acta *1837*, 2004–2016.
